# Demand-side barriers and economic burden in accessing Human Papillomavirus screening for cervical cancer prevention in rural India: Evidence from a cross-sectional study

**DOI:** 10.12688/f1000research.150361.1

**Published:** 2024-06-13

**Authors:** Shyamkumar Sriram, Arun Daniel Jayakumar, Pavan Kumar Gollapalli, Swetha Chandrasekar

**Affiliations:** 1 Department of Social and Public Health, Ohio University, Athens, Ohio, 45701, USA; 2 Department of Community Medicine, Aarupadai Veedu Medical College, Pondicherry, 607402, India; 3 Department of Community Medicine, Chettinad Academy of Research and Education, Kelambakkam, 603103, India; 4 Department of Obstetrics and Gynaecology, Shri Sathya Sai Medical College and Research Institute, Ammapettai, 603108, India

**Keywords:** HPV screening, cervical cancer prevention, rural India, healthcare accessibility, economic burden, healthcare costs

## Abstract

**Introduction:**

Cervical cancer is a significant global health concern, especially in low- and middle-income countries with limited access to preventive healthcare. India’s vast rural population amplifies the challenge, demanding immediate action. Despite advancements, cervical cancer remains prevalent among underserved rural communities, hindered by barriers to Human Papillomavirus (HPV) screening uptake, including socioeconomic and financial constraints. This study aims to evaluate the economic challenges encountered by rural women when accessing HPV screening.

**Methods:**

A cross-sectional survey was conducted among 1502 women aged 30 to 45 in Pondicherry, India, utilizing the Andersen Model as a conceptual framework. Household questionnaires gathered data on HPV screening expenses, including patient travel costs, productivity loss, and companion costs. The analysis utilized regression models, to identify the factors impacting the economic challenges associated with accessing HPV screening.

**Results:**

Employment status and higher education significantly increase total costs by 73.483 (p < 0.001) and 90.169 units (p < 0.001) respectively. Income level, though with a minimal coefficient (B = 0.000), shows a significant effect (p = 0.019) on total costs. Longer travel hours raise costs by 5.129 units (p < 0.001), while having a companion increases costs by 106.095 units (p = 0.004). Prolonged patient time at Primary Health Center (PHC) contributes to a 2.357-unit increase in costs (p < 0.001).

**Conclusions:**

The study highlights the multifaceted economic challenges faced by rural populations accessing HPV screening for cervical cancer prevention in India. Notwithstanding diverse demographics and varying proximity to healthcare facilities, individuals encounter significant barriers such as travel time and associated costs. Addressing these challenges necessitates targeted interventions to reduce socioeconomic disparities and improve healthcare accessibility for vulnerable populations, thereby advancing cervical cancer prevention efforts and promoting health equity in rural communities.

## Introduction

Cervical cancer remains a significant global health burden, particularly in low- and middle-income countries (LMICs) where access to preventive healthcare services is often limited. ^1^ As the fourth most prevalent cancer among women worldwide, it recorded approximately 660,000 new cases and 350,000 fatalities in 2022. ^2^

India harbours a substantial population of approximately 511.4 million women aged 15 years and older who are at risk of developing cervical cancer, emphasizing the pressing need to address this health challenge. ^3^ Annually, an estimated 123,907 women are diagnosed with cervical cancer, and 77,348 succumb to the disease. Cervical cancer ranks as the second most common cancer among Indian women, particularly those aged 15 to 44 years, exerting a profound impact nationwide. The prevalence of cervical Human Papillomavirus (HPV) – 16/18 infection among the general female population is estimated to be around 5.0%, with HPV types 16 or 18 accounting for approximately 83.2% of invasive cervical cancer cases. This data highlights the pivotal role of HPV vaccination and screening programs in combating the disease. ^4^

Although females make up slightly over 48% of India’s rural population, only 1.7% of rural women participated in cervical cancer screening according to data from the National Family Health Survey 5 (NFHS-5). Cervical cancer disproportionately affects rural areas where healthcare access is limited, and awareness of preventive measures is lacking. It is crucial to address these disparities in healthcare access and education to effectively reduce the impact of cervical cancer in India. ^5^

HPV screening has emerged as a promising tool for early detection and prevention of cervical cancer. However, the uptake of HPV screening services in rural India is hindered by a myriad of demand-side barriers, including socioeconomic challenges and the financial burdens linked to HPV screening. These include both direct costs, such as transportation fees, lost income due to missed work, and out-of-pocket expenditures for healthcare services, as well as indirect expenses. ^6^ India, with its vast rural population and diverse socio-cultural landscape, faces a particularly daunting burden of cervical cancer. ^7^ Nevertheless, advances in screening and prevention methods, the disease continues to exact a heavy toll, disproportionately affecting women in underserved rural communities. ^8^^,^^9^

HPV testing presents distinct advantages over traditional cytology-based methods like Pap smear, with higher sensitivity, lower false-negative rates, and the capability to detect HPV infection prior to cytological abnormalities, making it advantageous for cervical cancer prevention especially in resource-limited settings. ^10^

Regardless of the potential benefits of HPV screening, its uptake in rural India is hampered by a range of demand-side barriers that impede access to screening services and contribute to disparities in cervical cancer outcomes. ^11^ Socio-economic factors play a significant role in shaping access to healthcare services, including HPV screening, in rural India. Poverty, lack of health insurance, and financial constraints often limit women’s ability to seek preventive care, including cervical cancer screening. In many rural households, healthcare expenses are perceived as a luxury rather than a necessity, leading women to prioritize other household needs over their own health. ^12^

Moreover, the cost of HPV testing and follow-up procedures, such as colposcopy and biopsy, can be prohibitive for women in rural areas, particularly those belonging to marginalized communities. Even when screening services are available free of charge or at subsidized rates, indirect costs such as transportation and lost wages may pose significant barriers to utilization, especially for women residing in remote villages with limited access to healthcare facilities. ^13^

In rural areas, socioeconomic factors intertwine to create formidable financial barriers for women seeking HPV screening for cervical cancer prevention. ^14^ The direct costs associated with accessing HPV screening services, including transportation expenses, pose significant challenges for rural residents, particularly those in remote areas. Additionally, the necessity of taking time off work to travel to healthcare facilities results in lost wages for many hourly or daily wage earners, further exacerbating the financial burden. ^15^ Beyond tangible costs, intangible yet impactful indirect expenses such as the opportunity cost of forgoing work or household responsibilities and psychological stress also deter rural women from seeking screening. These financial burdens contribute to decreased utilization of preventive healthcare services among rural women, exacerbating existing health inequities. ^16^ Addressing these barriers requires a comprehensive approach encompassing policy reforms, targeted interventions, and community engagement strategies to ensure equitable access to cervical cancer screening services and improve the health outcomes of rural women globally. ^17^

**Figure 1.**
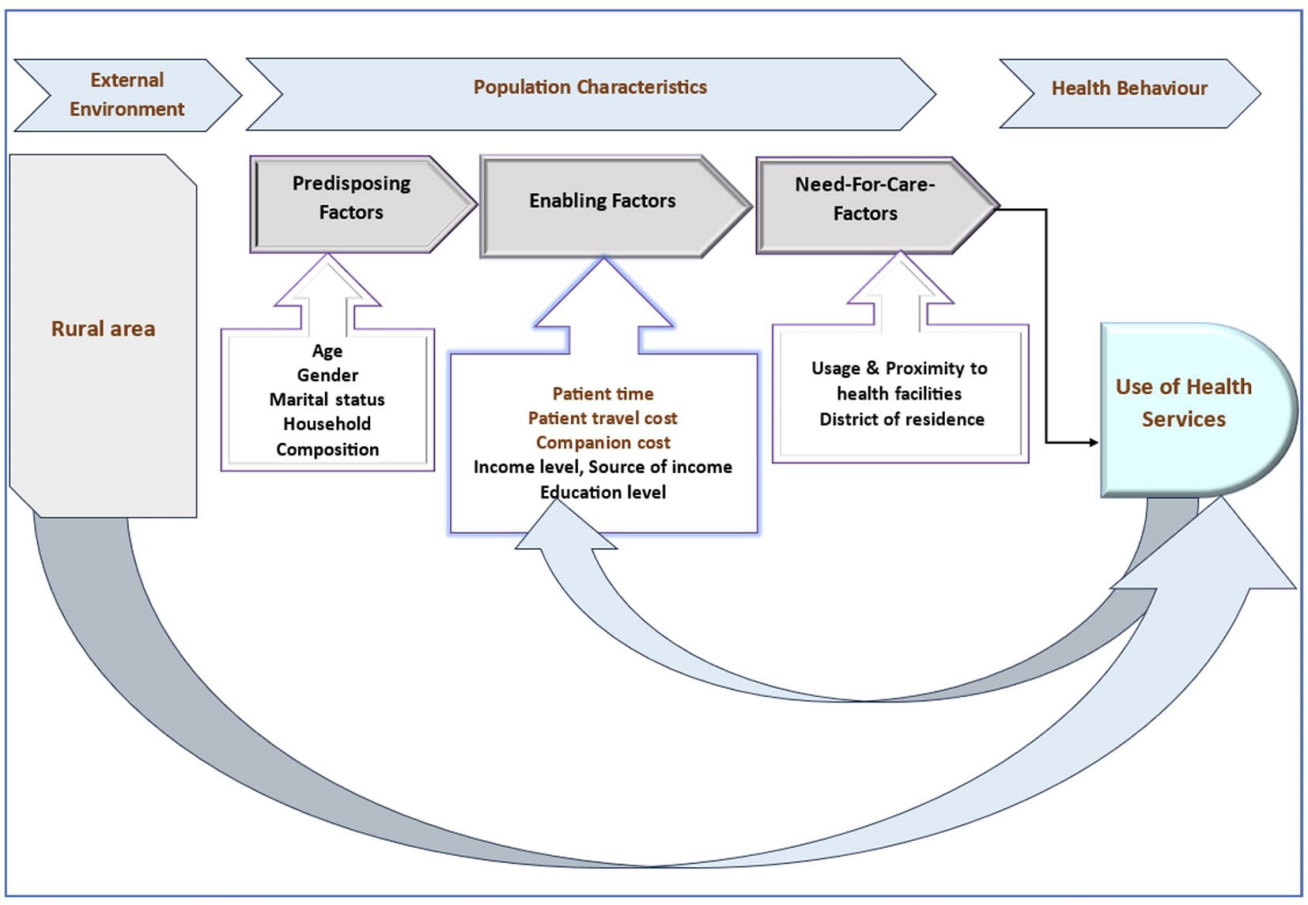
Conceptual framework for understanding healthcare access and utilization.

The conceptual framework ( Figure 1) for this study draws upon the Andersen Model, ^18^ a well-established framework in healthcare research. The Andersen Model emphasizes the interplay between predisposing factors, enabling resources, and need factors in shaping healthcare access and utilization. This model provides a comprehensive framework for understanding the various determinants of healthcare-seeking behaviour and utilization patterns.

**Predisposing factors:** These are characteristics that predispose individuals to seek or avoid healthcare services. In this study, predisposing factors include socio-demographic characteristics such as age, gender, marital status, and household composition.

**Enabling resources:** Enabling resources encompass economic aspects that facilitate or hinder healthcare access and utilization. This includes household consumption expenditures like travel costs, patient time, companion costs, childcare expenses, income level, source of income, and education level.

**Need factors:** Need factors represent the perceived or actual need for healthcare services. This includes healthcare payments, health status, usage patterns, proximity to health facilities, and district of residence.

Therefore, the study aims to analyse the economic challenges faced by rural Indian women when accessing HPV screening for cervical cancer prevention. It aims to quantify the costs involved, including transportation expenses, lost wages due to time off work, and other financial implications.

## Methods

This cross-sectional study was conducted among women aged 30 to 45 in Pondicherry, India. Pondicherry, being a Union Territory in India, was chosen as the study area due to its manageable size and diverse population of 8,98,000 individuals.

In the sampling strategy employed for the study, a total of 1500 women aged 30 to 45 were chosen randomly from the catchment areas of 15 selected Primary Health Centers (PHCs) in Pondicherry, India. It was estimated that each PHC catchment area covered approximately 30,000 individuals.

The sampling process unfolded across three stages. Initially, in stage 1, the PHCs were designated as primary sampling units, and a simple random sampling technique was utilized to select 15 PHCs from a list obtained from the District Public Health office. The selected PHCs included Abishegapakkam, Ariyankuppam, Bahour, Gorimedu, Karikalampakkam, Kirumambakkam, Koodapakkam, Mettupalayam, Nettapakkam, Reddiarpalayam, Thavalakuppam, Villianur, Ariyur, Sedarapet, and Karayamputhur.

Subsequently, in stage 2, the Anganwadi centers within the chosen PHCs were designated as secondary sampling units. Within each PHC, five Anganwadi centers (AWCs) were randomly selected to ensure a representative sample. Finally, in stage 3, women aged 30 to 45 were selected within the chosen Anganwadi centers. From each selected AWC, 20 women within the specified age range were sampled using simple random sampling from the beneficiary lists. This meticulous approach resulted in a total sample size of 1500 women, with 100 women sampled from each PHC catchment area, thereby ensuring a comprehensive representation of the target population for the study.

The Household Cost Questionnaire (HCQ) administered to sampled women encompasses a wide array of variables spanning, socio-demographic variables include age, gender, education level, income level, source of income, and district of residence. Data on patient travel expenses, time spent on travel, companion expenses, childcare expenses, and productivity loss was collected comprehensively to accurately reflect the costs incurred by patients and their companions.

Institutional Ethical approval was obtained from the Institutional Review Board of Ohio University. IRB project 23-E-101, titled ‘Supply-side and Demand-side Barriers to Access HPV Screening and the Cost-effectiveness analysis of Human Papilloma Virus (HPV) Screening for the Prevention of Cervical Cancer Screening in India. Ohio University’s Institutional Review Board deemed it exempt from review since no interventions were carried out.

### Operational definition

**Total cost incurred:** The sum of all expenses associated with accessing HPV testing, including patient travel costs, companion costs, childcare/dependent costs, and productivity loss costs. Calculated by adding up the costs from each category.

**Patient travel cost:** The expenses incurred by the patient for traveling to and from the PHC for HPV testing. Calculated separately for each mode of transportation used (public transport and taxi), considering round trip costs.

**Companion cost:** The expenses incurred by the companion who accompanies the patient to the PHC. Calculated by summing the cost of one-way fare for the companion’s travel and the amount of time taken off from paid work to accompany the patient.

**Productivity loss cost:** The monetary value of earnings lost by the patient due to time taken off from work to visit the PHC. Calculated based on the average salary of a person in India/Pondicherry multiplied by the amount of time taken off from work.

**Average salary of a person in India/Pondicherry:** The mean income earned by individuals in the specified geographical area. The average wage earning for rural women worker is Rs 265 per day (National Statistical Office 2022). ^19^

Firstly, the exposure variables included participants’ employment status, education level, and income level. Employment status was categorized as either employed or unemployed, while education level was assessed based on the highest level of education attained, ranging from primary to post-graduate university. Income level was determined by annual household income, segmented into different income brackets. These variables provided insights into the socio-economic status of the participants and their households, which are crucial factors influencing healthcare access and utilization.

Secondly, the primary outcome variable examined was the total healthcare costs incurred by participants when accessing HPV testing. These costs encompassed various components, including patient travel expenses, companion expenses, child care expenses, and productivity losses. By quantifying these costs, the study aimed to assess the economic burden experienced by participants and their companions during the process of seeking HPV testing services.

Additionally, several covariates were considered in the analysis to account for potential confounding factors. These covariates included age, gender, district of residence, source of income, distance from home to the PHC, hours travelled to the PHC, and whether a companion accompanied the participant during PHC visits. These factors were deemed relevant as they could influence both the exposure and outcome variables, thus requiring adjustment in the analysis to obtain accurate estimates of the associations under investigation.

Collected data was analysed using STATA 16. Descriptive statistics, such as means, medians, ranges, and standard deviations, are used to describe the demographic characteristics of the study population and variables related to healthcare access and utilization.

Individual costs were aggregated to estimate the total economic burden for patients. Total costs for the entire sample and average costs per patient were calculated. Regression models are employed to examine the relationship between independent variables (e.g., employment status, education level) and the dependent variable (total healthcare costs). This helps identify significant predictors of the economic burden of accessing healthcare services.

Institutional Ethical approval was obtained from the Institutional Review Board of Ohio University. Informed written consent was obtained from all study participants before data collection.

## Results

The demographic breakdown of the surveyed population ( Table 1) consisting of 1502 individuals, showcases a varied distribution across female age groups, with the highest percentage falling between 31 to 40 years (34.75%), closely followed by the 21 to 30 years range (30.89%). The majority of respondents are married (85.62%). Employment status displays diversity, with homemakers representing the largest segment (70.64%), followed by those engaged in full-time (10.19%) and part-time (13.05%) work. Education levels range from primary to post-graduate university, with a noteworthy proportion having attained some secondary education (32.42%). Annual household incomes comprise a significant proportion falling below 50,000 INR (28.03%). The majority of households accommodate four or fewer adults (86.82%) and two or fewer children (96.40%).

**Table 1.** Demographic characteristics of the study variables.

Variables	Categories	Frequency (N = 1502)	Percentage (%)
**Age in years**	15 to 20	108	7.19
	21 to 30	464	30.89
	31 to 40	522	34.75
	More than 40	408	27.16
**Marital status**	Single	195	12.98
	Married	1286	85.62
	Separated	5	0.33
	Divorced	2	0.13
	Widowed	11	0.73
	Other (Specify)	3	0.20
**Employment status**	In full-time work	153	10.19
	In part-time work	196	13.05
	Currently seeking work	26	1.73
	Homemaker	1061	70.64
	Retired	1	0.07
	Both in part-time & full-time	1	0.07
	Other (Specify)	64	4.26
**Education level**	All secondary	338	22.50
	College	297	19.77
	Post-graduate university	3	0.20
	Primary	351	23.37
	Primary, University	1	0.07
	Some secondary	487	32.42
	Some secondary University	1	0.07
	University	24	1.60
**Annual household income (INR)**	Less than 50,000 INR	421	28.03
	More than 50,000 INR & less than 100,000 INR	349	23.24
	More than 100,000 INR and less than 200,000 INR	416	27.70
	More than 200,000 INR	316	21.04
**Adults in household**	4 and less than 4 adults in household	1304	86.82
	More than 4 adults in household	198	13.18

Table 2 provides essential demographic and geographic variables pertaining to healthcare access. The mean annual household income is 155,560 INR, with a median of 100,000 INR and a substantial range spanning 6,000,000 INR. This discrepancy between the mean and median suggests a positively skewed distribution influenced by high-income outliers. Age distribution, with a mean of 34.08 years and a median of 34 years, appears relatively symmetric, indicating a balanced spread across age groups. The number of adults in households has a mean of 2.96 and a median of 3, with a range extending to 10, reflecting moderate variability in household composition. Similarly, the number of children in households shows a mean of 1.07 and a median of 1, with a range of 15, suggesting varied family sizes. Geographic metrics reveal wider disparities, with a mean distance from home to the Primary Health Center (PHC) of 3.64 kilometers and a median of 2 kilometers. This disparity between mean and median distances indicates significant variability, possibly reflecting urban-rural disparities in accessibility. Additionally, the distances traveled by private car or motorbike, reaching up to 299 km one-way, provide insights into transportation needs and possibly lifestyle preferences. Collectively, these data points infer a multifaceted picture of households, highlighting disparities in income, demographics, and geographic access, crucial for understanding and addressing diverse societal needs and challenges.

**Table 2.** Summary of household characteristics and geographic proximity to PHCs.

Variables	Mean	Median	Range	SD
**Annual Household Income (INR)**	155,560	100,000	6,000,000	202,103.80
**Age**	34.08	34	43	8.91
**Number of adults in household**	2.96	3	10	1.43
**Number of children in household**	1.07	1	15	0.99
**Distance from home to the PHC (km)**	3.64	2	500	15.39
**Distance traveled by private car or motorbike (one-way) (km)**	22.06	3	299	43.50

Table 3 presents frequencies and percentages related to various variables associated with households’ interactions with Primary Health Centers (PHCs). Notably, a significant majority of households, comprising 80.36%, reside within a 3-kilometer radius from a PHC, suggesting relatively close proximity for accessing healthcare services. However, a notable proportion, 15.85%, live farther, between 3 and 10 kilometers from the nearest PHC. Moreover, a smaller percentage, 2.60%, reside beyond 10 kilometers, indicating potential challenges in accessing healthcare for these households.

**Table 3.** Variables related to access and utilization of PHC services.

Variables	Categories	Frequency (N = 1502)	Percentage (%)
**Distance from home to the PHC (km)**	Less than or equal to 3 km	1207	80.36
	More than 3 km and less than 10 km	238	15.85
	More than 10 km and less than 20 km	39	2.60
	More than 10 minutes & less than 20 minutes	666	44.34
	More than 20 minutes & less than 30 minutes	151	10.05
**Travelled by public transport**	No	1255	83.56
	Yes	247	16.44
**Returned home using the same form of transport**	Yes	1414	94.14
	No	88	5.86
**Companion accompanied the person to the PHC**	Yes	186	12.38
	No	1316	87.62

In terms of travel duration, a considerable portion of individuals, accounting for 44.34%, reported travel times of more than 10 minutes but less than 20 minutes to reach the PHC, with 10.05% enduring journeys lasting between 20 and 30 minutes. This infers varying degrees of travel inconvenience potentially experienced by households when seeking healthcare.

Regarding transportation modes, a majority, comprising 83.56%, did not utilize public transport, suggesting a reliance on private means of transportation. Additionally, a significant majority, 94.14%, returned home using the same mode of transport, indicating consistency in transportation choices.

Furthermore, the data reveals insights into social dynamics, with only 12.38% of individuals being accompanied by a companion to the PHC. This indicates that for the majority, healthcare-seeking behaviour occurs independently.

Table 4 offers insights into various time-related aspects concerning individuals’ interactions with PHCs and related responsibilities.

**Table 4.** Factors influencing time management and work obligations during visits to PHCs.

Variables	Categories	Frequency (N = 1502)	Percentage (%)
**Time spent in the PHC (waiting time & consultation time)**	5 and less than 5 minutes	129	8.59
	More than 5 minutes & less than 10 minutes	122	8.12
	More than 10 minutes & less than 20 minutes	254	16.91
	More than 20 minutes & less than 60 minutes	964	64.18
	More than 60 minutes	33	2.20
**Time taken from paid work to come to the PHC (minutes)**	More than 10 mins & less than 30 mins	4	6.35
	More than 30 mins & less than 60 mins	21	33.33
**Number of days in a week, an individual works**	Less than 4 days	4	3.51
	More than 4 days & less than 5 days	39	34.21
	More than 5 days & less than 7 days	71	62.28
**Number of hours in a week, on average, the individual works (hours)**	10 hours and less than 10 hours per week	44	38.60
	More than 10 hours and less than 20 hours per week	6	5.26
	More than 20 hours and less than 40 hours per week	37	32.46
	More than 40 hours and less than 72 hours per week	27	23.68
**Time taken from work to visit the Primary Health Center (minutes)**	10 minutes or less	27	23.68
	More than 10 mins & less than 30 mins	33	28.95
	More than 30 mins & less than 60 mins	38	33.33
	More than 60 mins & less than 120 mins	16	14.04
**Time spent by the companion both in travel time and time spent at the PHC (minutes)**	10 minutes or less	50	26.88
	More than 10 mins & less than 30 mins	41	22.04
	More than 30 mins & less than 60 mins	74	39.78
	More than 60 mins & less than 120 mins	21	11.29
**Amount of time taken off from paid work to accompany the individual to the PHC (minutes)**	10 minutes or less	149	80.11
	More than 10 mins & less than 30 mins	6	3.23
	More than 30 mins & less than 60 mins	13	6.99
	More than 60 mins & less than 120 mins	12	6.45
	More than 120 mins & less than 300 mins	6	3.23
**Time spent looking after the children/dependents by the caregiver when the individual visited the PHC**	15 minutes or less	22	34.92
	More than 15 mins & less than 30 mins	16	25.40
	More than 30 mins & less than 60 mins	21	33.33
	More than 60 mins & less than 120 mins	4	6.35

Firstly, concerning the time spent at the PHC, the majority of individuals, constituting 64.18%, reported durations of more than 20 minutes but less than 60 minutes, emphasizing potentially significant waiting and consultation times. Moreover, 8.12% and 16.91% experienced shorter durations, while a smaller proportion, 2.20%, endured waits exceeding 60 minutes.

In terms of time allocation from paid work to visit the PHC, there’s a distribution across various durations, with 33.33% spending more than 30 minutes but less than 60 minutes, indicating potential disruptions to work schedules for healthcare visits.

Furthermore, data regarding work schedules reveals that the majority, comprising 62.28%, work more than 5 days but less than 7 days per week, underscoring potential challenges in balancing work commitments with healthcare needs.

Regarding time spent by companions, there’s variability, with 39.78% spending more than 30 minutes but less than 60 minutes, possibly reflecting the support provided by companions in accompanying individuals to PHCs.

Moreover, data on time taken off from paid work to accompany individuals to PHCs highlights that a significant majority, at 80.11%, reported durations of 10 minutes or less, indicating minimal disruptions to work for caregiving responsibilities.

Lastly, concerning caregiving responsibilities, caregivers spent varying durations looking after children/dependents during individuals’ visits to PHCs, with 33.33% spending more than 30 minutes but less than 60 minutes, reflecting the impact of healthcare visits on caregiving duties.

Overall, these insights shed light on the time-related challenges and dynamics individuals and their companions face when accessing healthcare services, highlighting areas where interventions or improvements may be necessary to streamline processes and reduce burdens on individuals and their support networks.

Table 5 presents a detailed overview of various time-related factors associated with individuals’ engagements with PHCs and their corresponding duties.

**Table 5.** Time-related challenges that the individuals and their companions encounter when accessing healthcare services.

Variables	Mean	Median	Range	SD
**Time taken to travel from home to the PHC (minutes)**	16.12	15	70	9.84
**Time taken from paid work to come to the PHC (minutes)**	52.52	60	120	45.18
**Time spent by the companion both in travel time and time spent at the PHC (minutes)**	36.18	40	120	27.44
**Amount of time taken off from paid work to accompany the individual to the PHC (minutes)**	62.05	47.50	299	67.13
**Time spent looking after the children/dependents by the caregiver when the individual visited the PHC (minutes)**	30.30	30	120	28.03
**Time taken from work to visit the Primary Health Center (minutes)**	41.71	30	120	35.81

For the duration of travel from home to the PHC, the mean time is 16.12 minutes, with a median of 15 minutes, indicating generally consistent travel times for most individuals. However, there is notable variability, with travel durations ranging from 0 to 70 minutes, and a standard deviation of 9.84 suggests moderate dispersion around the mean.

Regarding the time taken from paid work to reach the PHC, the mean duration is higher at 52.52 minutes, with a median of 60 minutes, reflecting potentially longer commutes for those traveling from their workplaces. The range spans from 0 to 120 minutes, indicating diverse commuting times, with a considerable standard deviation of 45.18.

Companions’ time commitments, including travel and time spent at the PHC, show a mean duration of 36.18 minutes, with a median of 40 minutes, suggesting moderate consistency. However, there is variability, with durations ranging from 0 to 120 minutes and a standard deviation of 27.44.

Individuals taking time off from work to accompany others to the PHC experience a mean duration of 62.05 minutes, with a median of 47.50 minutes, highlighting significant disruptions to work schedules. The range is wide, from 0 to 299 minutes, with a considerable standard deviation of 67.13.

Lastly, caregivers spend an average of 30.30 minutes looking after children/dependents during PHC visits, with a median of 30 minutes, showcasing consistent caregiving responsibilities. Variability exists, with durations ranging from 0 to 120 minutes and a standard deviation of 28.03.

Table 6 presents detailed insights into the various costs and time implications associated with patient travel, companion expenses, childcare, and productivity losses related to visits to the PHC.

**Table 6.** Patient’s comprehensive expenses for rural PHC treatment access.

Variables	Mean	Median	Range	SD
**Patient travel costs**				
Cost of one-way fare if traveled by public transport (INR)	352.34	20	79999	2064.14
Cost of one-way taxi fare (INR)	58.46	50	198	37.64
Cost of tolls if travel by private car or motorbike (INR)	52.42	50	199	39.94
**Patient time costs**				
Amount of earnings lost due to time taken off work to go to the PHC (INR)	99.20	50	500	129.35
**Companion costs**				
Cost of one-way fare if the companion traveled by public transport with the patient (INR)	54.60	50	298	56.79
**Childcare and other dependent costs**				
Amount paid to that person to look after children/dependents when the individual visited the PHC (INR)	3.66	0	20	8.04
**Productivity losses**				
Number of days a week individual works	5.06	6	6	0.74
Number of hours a week, on average the individual works (hours)	27.23	34	70	18.50

Public transport one-way fares exhibit a wide range, spanning from 20 INR to 79,999 INR, reflecting various factors such as distance and service types. Similarly, taxi fares for single journeys display notable variability, ranging from 37.64 INR to 198 INR. Toll expenses for private vehicles, meanwhile, range from 39.94 INR to 199 INR, highlighting differences in toll rates influenced by factors like distance and road infrastructure.

For patients, the cost encompasses the income lost from taking time off work to visit a PHC, ranging from 50 INR to 129.35 INR. When patients are accompanied, additional expenses arise, such as one-way transport fares for companions, ranging from 50 INR to 56.79 INR. Furthermore, there are costs associated with childcare or dependent care during the patient’s absence, varying from 0 INR to 8.04 INR.

In terms of productivity losses, the data reflects the number of workdays missed per week (ranging from 6 to 6.74) and the average weekly working hours (ranging from 34 to 70 hours). These figures illustrate the impact of healthcare visits on individuals’ work commitments and earnings potential.

Table 7 revealed that the employed individuals experience an increase in total costs by 73.483 units (95% CI: 38.81-108.153) when accessing HPV screening. The p-value being less than 0.001 suggests that this association is statistically significant. This implies that employment status is a significant predictor of the economic burden of accessing HPV screening in rural India.

**Table 7.** Factors impacting total healthcare costs: Insights from Regression analysis.

Variables	Unstandardized Coefficients		Stand. Coef. (B)	95% Confidence Interval	P
	B	Std. Error			
**Intercept**	-286.122	65.238		-414.089 - -158.154	0.000
**Employment status**	73.483	17.675	0.107	38.81-108.153	0.000
**Education level**	90.169	19.444	0.125	52.030-128.309	0.000
**Income level**	0.000	0.000	0.062	.000-.000	0.019
**Distance from home to PHC**	-0.158	.787	-0.005	-1.702-1.386	0.841
**Hours travelled to PHC**	5.129	1.278	0.104	2.622-7.635	0.000
**Companion accompanied during PHC visits**	106.095	37.123	0.072	33.276-178.913	0.004
**Patient time in PHC**	2.357	0.560	0.112	1.258-3.455	0.000

Higher education levels are associated with higher total costs, with an increase of 90.169 units (95% CI: 52.030-128.309). The p-value being less than 0.001 indicates statistical significance, suggesting that education level plays a significant role in determining the economic burden of accessing HPV screening in rural India.

Income level shows a statistically significant effect (p = 0.019) on total costs, although the coefficient is negligible (B = 0.000). This implies that while income level may have a minimal impact, it still contributes to the economic burden of accessing HPV screening, as indicated by the 95% confidence interval.

The distance from home to the PHC does not significantly impact total costs (p = 0.841). The coefficient of -0.158 suggests a minor decrease in total costs with an increase in distance, but this effect is not statistically significant, as indicated by the wide 95% confidence interval.

Longer travel hours to the PHC significantly increase total costs by 5.129 units (95% CI: 2.622-7.635). The p-value being less than 0.001 indicates statistical significance, highlighting the importance of travel time as a determinant of the economic burden of accessing HPV screening.

Having a companion during PHC visits significantly increases total costs by 106.095 units (95% CI: 33.276-178.913, p = 0.004). This suggests that the presence of a companion adds to the economic burden of accessing HPV screening in rural India.

Longer patient time in the PHC significantly increases total costs by 2.357 units (95% CI: 1.258-3.455, p < 0.001). This underscores the impact of extended waiting or consultation times on the economic burden of accessing HPV screening in rural India.

## Discussion

The study emphasizes the significant impact of socioeconomic factors on healthcare accessibility and affordability. Variables like employment status, educational attainment, and income level are pivotal in determining the financial strain associated with accessing HPV screening services. Those with higher socioeconomic status typically face fewer obstacles due to their greater financial means and enhanced access to healthcare facilities. The specific finding that employment status is a significant predictor of the economic hurdles in accessing HPV screening in rural India highlights the intricate interplay between socioeconomic factors and healthcare utilization. This observation is in line with the research conducted by Srivatsa et al., which suggests that women hailing from households with a higher income are significantly more inclined to undergo cervical cancer screening compared to those from lower-income households. ^20^^,^^21^ Additional studies such as Kaneko, 2018, and Keetile et al., 2021 have similarly argued that disadvantaged households are often less informed and thus less likely to prioritize cervical cancer screening. ^22^^,^^23^

A notable finding underlines the influence of travel-related variables on overall expenses. Extended travel duration to the PHC and having a companion during PHC visits are associated with increased total costs. These results are consistent with prior research conducted by Rocque (2019) and Kornelson (2021), ^24^^,^^25^ highlighting the significant contribution of travel-related expenses, to the economic burden experienced by individuals accessing HPV screening in rural areas. This observation is further supported by Wu et al. (2020) and Srinath et al (2023). ^26^^,^^27^ Addressing transportation barriers and providing assistance for travel expenses could prove instrumental in easing the economic burden on vulnerable populations. ^28^^,^^29^

The study underscores the significance of healthcare delivery efficiency in mitigating the economic burden on patients. Prolonged patient time in PHC facilities correlates positively with increased total costs, underscoring the need for streamlined healthcare processes to minimize waiting times and optimize resource utilization. Improvements in healthcare infrastructure and the implementation of efficient appointment systems can enhance the quality and accessibility of HPV screening services while reducing costs for patients. Shyam et al.’s findings revealed that for-profit hospitals tend to have shorter waiting times for patients, potentially attracting greater demand from economically advantaged individuals. This emphasizes the importance of reducing waiting times in public sector hospitals to ensure equitable access to healthcare services across all socioeconomic strata. Furthermore, considering the sociodemographic and community-level factors, providers can better strategize to improve screening uptake within their local practice settings. ^30^^–^^32^

Initiatives to enhance access to HPV screening should not only address geographical barriers but also consider the socioeconomic determinants that may deter individuals from seeking preventive care. By addressing these disparities, policymakers and healthcare providers can strive toward ensuring equitable access to vital healthcare services, thereby alleviating the burden of preventable diseases like cervical cancer in rural India and beyond. ^4^^,^^15^^,^^33^

The findings of the study have important policy implications for cervical cancer prevention efforts in rural India. Policy interventions aimed at improving employment opportunities, promoting education, and enhancing transportation infrastructure can help alleviate the socioeconomic barriers to accessing HPV screening services. Additionally, targeted financial assistance programs for low-income individuals and those living in remote areas can help reduce the economic burden associated with seeking healthcare services. ^11^^,^^34^

The study acknowledges several limitations, such as its cross-sectional design and potential confounding factors. Future research could explore longitudinal data to assess the long-term economic impact of accessing HPV screening and investigate additional factors influencing the economic burden, such as out-of-pocket expenses and indirect costs. Moreover, qualitative studies could provide deeper insights into the lived experiences of individuals accessing HPV screening services and the factors influencing their decision-making processes.

In conclusion, the article contributes to our understanding of the economic challenges faced by individuals accessing HPV screening for cervical cancer prevention in rural India. By identifying key socioeconomic factors and travel-related costs influencing total costs, the study provides valuable insights for policymakers and healthcare providers to develop targeted interventions aimed at reducing the economic burden and improving healthcare access for vulnerable populations.

## Ethics approval and consent to participate

Institutional Ethical approval was obtained from the Institutional Review Board of Ohio University on 05.17.2023. IRB project 23-E-101, titled ‘Supply-side and Demand-side Barriers to Access HPV Screening and the Cost-effectiveness analysis of Human Papilloma Virus (HPV) Screening for the Prevention of Cervical Cancer Screening in India. Ohio University’s Institutional Review Board deemed it exempt from review since no interventions were carried out.

Informed written consent was obtained from all study participants before data collection.

## Consent for publication

Not applicable.

## Data Availability

**Harvard Dataverse**: Demand-side Barriers and Economic Burden in Accessing Human Papillomavirus Screening for Cervical Cancer Prevention in Rural India: Evidence from a Cross-sectional Study, https://doi.org/10.7910/DVN/H9DB7B. ^35^ This project contains the following underlying data:
- Microsoft Excel Spreadsheet Data are available under the terms of the Creative Commons Zero “No rights reserved” data waiver (CC0 1.0 Public domain dedication) Harvard Dataverse: Demand-side Barriers and Economic Burden in Accessing Human Papillomavirus Screening for Cervical Cancer Prevention in Rural India: Evidence from a Cross-sectional Study, https://doi.org/10.7910/DVN/H9DB7B. ^35^ This project contains the following extended data:
- Questionnaire Data are available under the terms of the Creative Commons Zero “No rights reserved” data waiver (CC0 1.0 Public domain dedication).
