# Demand-side barriers and economic burden in accessing Human Papillomavirus screening for cervical cancer prevention in rural India: Evidence from a cross-sectional study

**DOI:** 10.12688/f1000research.150361.2

**Published:** 2025-02-17

**Authors:** Shyamkumar Sriram, Arun Daniel Jayakumar, Pavan Kumar Gollapalli, Swetha Chandrasekar

**Affiliations:** 1 Department of Social and Public Health, Ohio University, Athens, Ohio, 45701, USA; 2 Department of Rehabilitation and Health Services, College of Health and Public Service, University of North Texas, Denton, Texas, USA; 3 Department of Community Medicine, Aarupadai Veedu Medical College, Pondicherry, 607402, India; 4 Department of Community Medicine, Chettinad Academy of Research and Education, Kelambakkam, 603103, India; 5 Department of Obstetrics and Gynaecology, Shri Sathya Sai Medical College and Research Institute, Ammapettai, 603108, India

**Keywords:** HPV screening, cervical cancer prevention, rural India, healthcare accessibility, economic burden, healthcare costs

## Abstract

**Introduction:**

Cervical cancer is a significant global health concern, especially in low- and middle-income countries with limited access to preventive healthcare. India’s vast rural population amplifies the challenge, demanding immediate action. Despite advancements, cervical cancer remains prevalent among underserved rural communities, hindered by barriers to Human Papillomavirus (HPV) screening uptake, including socioeconomic and financial constraints. This study aims to evaluate the economic challenges encountered by rural women when accessing HPV screening.

**Methods:**

A cross-sectional survey was conducted among 1502 women aged 30 to 45 in Pondicherry, India, utilizing the Andersen Model as a conceptual framework. Household questionnaires gathered data on HPV screening expenses, including patient travel costs, productivity loss, and companion costs. The analysis utilized regression models, to identify the factors impacting the economic challenges associated with accessing HPV screening.

**Results:**

The study found that employment status significantly influenced healthcare costs, with employed women incurring ₹65.78 more than unemployed women (p < 0.001). Education level was also a significant predictor, with each additional year of education leading to a ₹108.45 increase in costs (p < 0.001). Travel time had a positive association with healthcare costs, with every additional minute spent traveling increasing costs by ₹5.98 (p < 0.001). Income and companion accompaniment were also significant predictors, while distance to the PHC and age did not show significant associations with total healthcare.

**Conclusions:**

The study highlights the multifaceted economic challenges faced by rural populations accessing HPV screening for cervical cancer prevention in India. Notwithstanding diverse demographics and varying proximity to healthcare facilities, individuals encounter significant barriers such as travel time and associated costs. Addressing these challenges necessitates targeted interventions to reduce socioeconomic disparities and improve healthcare accessibility for vulnerable populations, thereby advancing cervical cancer prevention efforts and promoting health equity in rural communities.

## Introduction

Cervical cancer remains a significant global health burden, particularly in low- and middle-income countries (LMICs) where access to preventive healthcare services is often limited. ^1^ As the fourth most prevalent cancer among women worldwide, it recorded approximately 660,000 new cases and 350,000 fatalities in 2022. ^2^

India harbours a substantial population of approximately 511.4 million women aged 15 years and older who are at risk of developing cervical cancer, emphasizing the pressing need to address this health challenge. ^3^ Annually, an estimated 123,907 women are diagnosed with cervical cancer, and 77,348 succumb to the disease. Cervical cancer ranks as the second most common cancer among Indian women, particularly those aged 15 to 44 years, exerting a profound impact nationwide. The prevalence of cervical Human Papillomavirus (HPV) – 16/18 infection among the general female population is estimated to be around 5.0%, with HPV types 16 or 18 accounting for approximately 83.2% of invasive cervical cancer cases. This data highlights the pivotal role of HPV vaccination and screening programs in combating the disease. ^4^

Although females make up slightly over 48% of India’s rural population, only 1.7% of rural women participated in cervical cancer screening according to data from the National Family Health Survey 5 (NFHS-5). Cervical cancer disproportionately affects rural areas where healthcare access is limited, and awareness of preventive measures is lacking. It is crucial to address these disparities in healthcare access and education to effectively reduce the impact of cervical cancer in India. ^5^

HPV screening has emerged as a promising tool for early detection and prevention of cervical cancer. However, the uptake of HPV screening services in rural India is hindered by a myriad of demand-side barriers, including socioeconomic challenges and the financial burdens linked to HPV screening. These include both direct costs, such as transportation fees, lost income due to missed work, and out-of-pocket expenditures for healthcare services, as well as indirect expenses. ^6^ India, with its vast rural population and diverse socio-cultural landscape, faces a particularly daunting burden of cervical cancer. ^7^ Nevertheless, advances in screening and prevention methods, the disease continues to exact a heavy toll, disproportionately affecting women in underserved rural communities. ^8^^,^^9^

HPV testing presents distinct advantages over traditional cytology-based methods like Pap smear, with higher sensitivity, lower false-negative rates, and the capability to detect HPV infection prior to cytological abnormalities, making it advantageous for cervical cancer prevention especially in resource-limited settings. ^10^

Regardless of the potential benefits of HPV screening, its uptake in rural India is hampered by a range of demand-side barriers that impede access to screening services and contribute to disparities in cervical cancer outcomes. ^11^ Socio-economic factors play a significant role in shaping access to healthcare services, including HPV screening, in rural India. Poverty, lack of health insurance, and financial constraints often limit women’s ability to seek preventive care, including cervical cancer screening. In many rural households, healthcare expenses are perceived as a luxury rather than a necessity, leading women to prioritize other household needs over their own health. ^12^

Moreover, the cost of HPV testing and follow-up procedures, such as colposcopy and biopsy, can be prohibitive for women in rural areas, particularly those belonging to marginalized communities. Even when screening services are available free of charge or at subsidized rates, indirect costs such as transportation and lost wages may pose significant barriers to utilization, especially for women residing in remote villages with limited access to healthcare facilities. ^13^

In rural areas, socioeconomic factors intertwine to create formidable financial barriers for women seeking HPV screening for cervical cancer prevention. ^14^ The direct costs associated with accessing HPV screening services, including transportation expenses, pose significant challenges for rural residents, particularly those in remote areas. Additionally, the necessity of taking time off work to travel to healthcare facilities results in lost wages for many hourly or daily wage earners, further exacerbating the financial burden. ^15^ Beyond tangible costs, intangible yet impactful indirect expenses such as the opportunity cost of forgoing work or household responsibilities and psychological stress also deter rural women from seeking screening. These financial burdens contribute to decreased utilization of preventive healthcare services among rural women, exacerbating existing health inequities. ^16^ Addressing these barriers requires a comprehensive approach encompassing policy reforms, targeted interventions, and community engagement strategies to ensure equitable access to cervical cancer screening services and improve the health outcomes of rural women globally. ^17^

**Figure 1.**
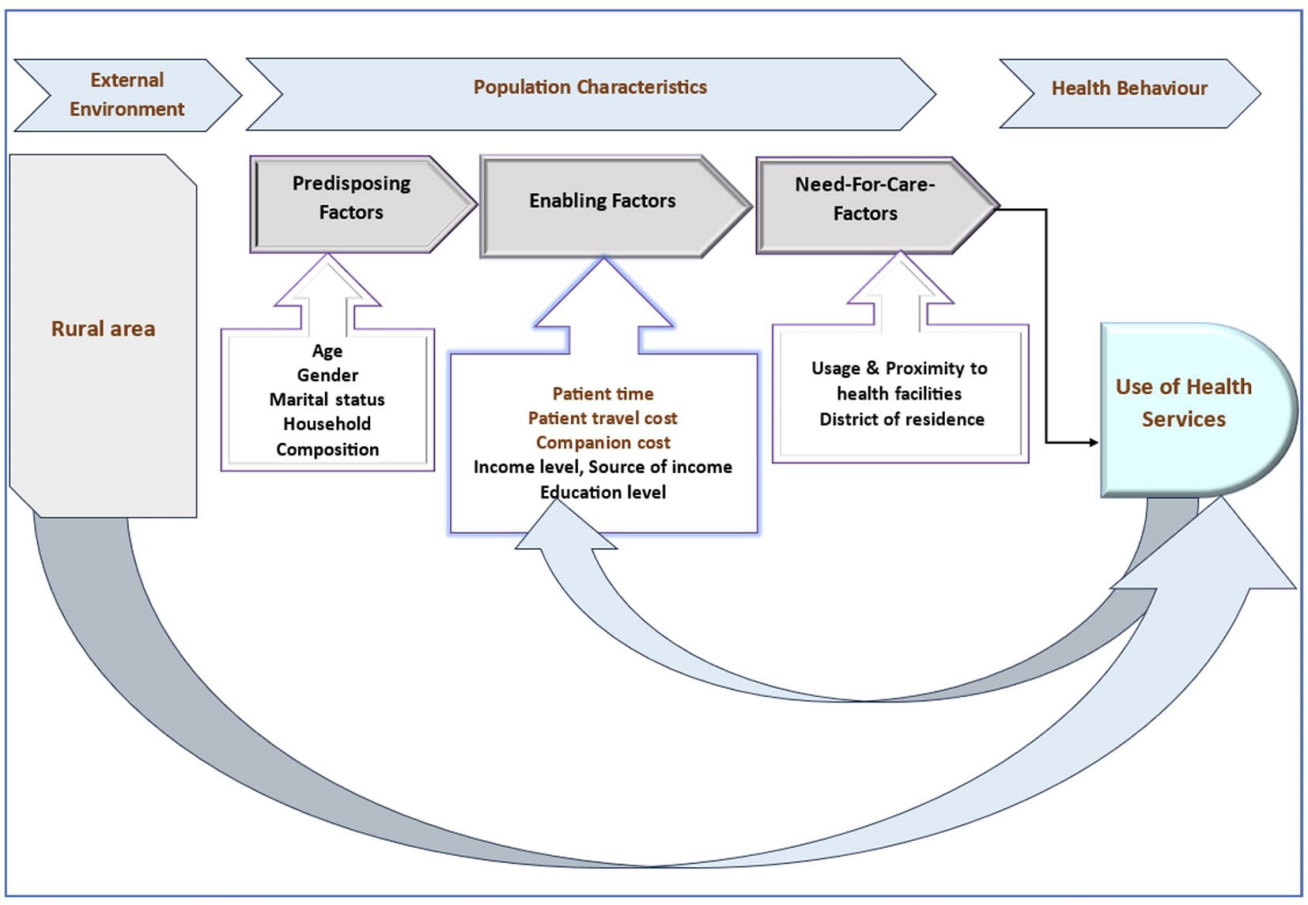
Conceptual framework for understanding healthcare access and utilization.

The conceptual framework ( Figure 1) for this study draws upon the Andersen Model, ^18^ a well-established framework in healthcare research. The Andersen Model emphasizes the interplay between predisposing factors, enabling resources, and need factors in shaping healthcare access and utilization. This model provides a comprehensive framework for understanding the various determinants of healthcare-seeking behaviour and utilization patterns.

**Predisposing factors:** These are characteristics that predispose individuals to seek or avoid healthcare services. In this study, predisposing factors include socio-demographic characteristics such as age, gender, marital status, and household composition.

**Enabling resources:** Enabling resources encompass economic aspects that facilitate or hinder healthcare access and utilization. This includes household consumption expenditures like travel costs, patient time, companion costs, childcare expenses, income level, source of income, and education level.

**Need factors:** Need factors represent the perceived or actual need for healthcare services. This includes healthcare payments, health status, usage patterns, proximity to health facilities, and district of residence.

Therefore, the study aims to analyse the economic challenges faced by rural Indian women when accessing HPV screening for cervical cancer prevention. It aims to quantify the costs involved, including transportation expenses, lost wages due to time off work, and other financial implications.

## Methods

**Study design:** This was a cross-sectional study conducted among women aged 30 to 45 years in Pondicherry, India.

**Study setting and participants:** Pondicherry, a Union Territory of India, was selected as the study area due to its manageable size and diverse population of approximately 898,000 individuals. The study population consisted of women aged 30 to 45 years residing within the catchment areas of selected Primary Health Centers (PHCs) in Pondicherry.

**Sampling strategy:** A three-stage random sampling method was used to select 1,500 participants from 15 PHCs in Pondicherry.

• **Stage 1:** A list of all PHCs in Pondicherry was obtained from the District Public Health Office. Fifteen PHCs were randomly selected using simple random sampling. The selected PHCs included Abishegapakkam, Ariyankuppam, Bahour, Gorimedu, Karikalampakkam, Kirumambakkam, Koodapakkam, Mettupalayam, Nettapakkam, Reddiarpalayam, Thavalakuppam, Villianur, Ariyur, Sedarapet, and Karayamputhur.
• **Stage 2:** Within each selected PHC, five Anganwadi Centers (AWCs) were randomly chosen.
• **Stage 3:** From each selected AWC, 20 women aged 30 to 45 years were randomly sampled from beneficiary lists, resulting in 100 women per PHC and a total sample size of 1,500 women.

**Data collection:** Data were collected using the Household Cost Questionnaire (HCQ), which captured socio-demographic variables such as age, education level, income level, source of income, and district of residence. Healthcare cost-related data were also collected, including patient travel expenses, time spent on travel, companion expenses, childcare expenses, and productivity losses.

**Exposure and outcome variables:**

• **Exposure variables:** Employment status (employed/unemployed), education level (ranging from primary education to postgraduate level), and income level (categorized into different household income brackets).
• **Outcome variable:** Total healthcare costs incurred for accessing HPV screening, including:
  ○ Patient travel expenses (round-trip costs for various transportation modes)
  ○ Companion expenses (travel costs and time off work)
  ○ Childcare expenses (costs for dependent care during the visit)
  ○ Productivity loss (earnings lost due to time off work, calculated based on the average daily wage of ₹265 for rural female workers, as per the National Statistical Office, 2022) ^19^

**Covariates and bias handling:** To minimize bias and improve the validity of findings, several methodological safeguards were implemented:

• **Selection bias:** A three-stage random sampling method ensured that participants were chosen randomly from eligible women within the PHC catchment areas, enhancing representativeness and reducing systematic differences.
• **Information bias:** Data were collected using a standardized HCQ administered by trained interviewers to ensure consistency and minimize recall bias. Travel cost estimates were cross-validated with local fare structures to improve data accuracy.
• **Confounding control:** Covariates such as age, distance from home to the PHC, travel time, and whether a companion accompanied the participant were included in the multivariable regression analysis to control for confounding factors.
• **Data quality and outlier management:** Extreme values in cost-related variables were examined, and any inconsistencies were addressed before analysis to ensure data reliability.

**Ethical considerations:** Institutional ethical approval was obtained from the Institutional Review Board of Ohio University (IRB project 23-E-101), titled *Supply-side and Demand-side Barriers to Access HPV Screening and the Cost-effectiveness Analysis of Human Papillomavirus (HPV) Screening for the Prevention of Cervical Cancer Screening in India*. The study was deemed exempt from review as no interventions were conducted.

**Data analysis:** Data were analyzed using STATA 16. Descriptive statistics (means, medians, standard deviations, and ranges) were used to summarize demographic characteristics and healthcare access variables. To assess the association between exposure variables (employment status, education level, and income level) and total healthcare costs incurred for HPV screening, multivariable regression models were employed.

## Results

The demographic breakdown of the surveyed population ( Table 1) consisting of 1502 individuals, showcases a varied distribution across female age groups, with the highest percentage falling between 31 to 40 years (34.75%), closely followed by the 21 to 30 years range (30.89%). The majority of respondents are married (85.62%). Employment status displays diversity, with homemakers representing the largest segment (70.64%), followed by those engaged in full-time (10.19%) and part-time (13.05%) work. Education levels range from primary to post-graduate university, with a noteworthy proportion having attained some secondary education (32.42%). Annual household incomes comprise a significant proportion falling below 50,000 INR (28.03%). The majority of households accommodate four or fewer adults (86.82%) and two or fewer children (96.40%).

**Table 1.** Demographic characteristics of the study variables.

Variables	Categories	Frequency (N = 1502)	Percentage (%)
**Age in years**	15 to 20	108	7.19
	21 to 30	464	30.89
	31 to 40	522	34.75
	More than 40	408	27.16
**Marital status**	Single	195	12.98
	Married	1286	85.62
	Separated	5	0.33
	Divorced	2	0.13
	Widowed	11	0.73
	Other (Specify)	3	0.20
**Employment status**	In full-time work	153	10.19
	In part-time work	196	13.05
	Currently seeking work	26	1.73
	Homemaker	1061	70.64
	Retired	1	0.07
	Both in part-time & full-time	1	0.07
	Other (Specify)	64	4.26
**Education level**	All secondary	338	22.50
	College	297	19.77
	Post-graduate university	3	0.20
	Primary	351	23.37
	Primary, University	1	0.07
	Some secondary	487	32.42
	Some secondary University	1	0.07
	University	24	1.60
**Annual household income (INR)**	Less than 50,000 INR	421	28.03
	More than 50,000 INR & less than 100,000 INR	349	23.24
	More than 100,000 INR and less than 200,000 INR	416	27.70
	More than 200,000 INR	316	21.04
**Adults in household**	4 and less than 4 adults in household	1304	86.82
	More than 4 adults in household	198	13.18

Table 2 provides essential demographic and geographic variables pertaining to healthcare access. The mean annual household income is 155,560 INR, with a median of 100,000 INR and a substantial range spanning 6,000,000 INR. This discrepancy between the mean and median suggests a positively skewed distribution influenced by high-income outliers. Age distribution, with a mean of 34.08 years and a median of 34 years, appears relatively symmetric, indicating a balanced spread across age groups. The number of adults in households has a mean of 2.96 and a median of 3, with a range extending to 10, reflecting moderate variability in household composition. Similarly, the number of children in households shows a mean of 1.07 and a median of 1, with a range of 15, suggesting varied family sizes. Geographic metrics reveal wider disparities, with a mean distance from home to the Primary Health Center (PHC) of 3.64 kilometers and a median of 2 kilometers. This disparity between mean and median distances indicates significant variability, possibly reflecting urban-rural disparities in accessibility. Additionally, the distances traveled by private car or motorbike, reaching up to 299 km one-way, provide insights into transportation needs and possibly lifestyle preferences. Collectively, these data points infer a multifaceted picture of households, highlighting disparities in income, demographics, and geographic access, crucial for understanding and addressing diverse societal needs and challenges.

**Table 2.** Summary of household characteristics and geographic proximity to PHCs.

Variables	Mean	Median	Range	SD
**Annual Household Income (INR)**	155,560	100,000	6,000,000	202,103.80
**Age**	34.08	34	43	8.91
**Number of adults in household**	2.96	3	10	1.43
**Number of children in household**	1.07	1	15	0.99
**Distance from home to the PHC (km)**	3.64	2	500	15.39
**Distance traveled by private car or motorbike (one-way) (km)**	22.06	3	299	43.50

Table 3 presents frequencies and percentages related to various variables associated with households’ interactions with Primary Health Centers (PHCs). Notably, a significant majority of households, comprising 80.36%, reside within a 3-kilometer radius from a PHC, suggesting relatively close proximity for accessing healthcare services. However, a notable proportion, 15.85%, live farther, between 3 and 10 kilometers from the nearest PHC. Moreover, a smaller percentage, 2.60%, reside beyond 10 kilometers, indicating potential challenges in accessing healthcare for these households.

**Table 3.** Variables related to access and utilization of PHC services.

Variables	Categories	Frequency (N = 1502)	Percentage (%)
**Distance from home to the PHC (km)**	Less than or equal to 3 km	1207	80.36
	More than 3 km and less than 10 km	238	15.85
	More than 10 km and less than 20 km	39	2.60
	More than 10 minutes & less than 20 minutes	666	44.34
	More than 20 minutes & less than 30 minutes	151	10.05
**Travelled by public transport**	No	1255	83.56
	Yes	247	16.44
**Returned home using the same form of transport**	Yes	1414	94.14
	No	88	5.86
**Companion accompanied the person to the PHC**	Yes	186	12.38
	No	1316	87.62

In terms of travel duration, a considerable portion of individuals, accounting for 44.34%, reported travel times of more than 10 minutes but less than 20 minutes to reach the PHC, with 10.05% enduring journeys lasting between 20 and 30 minutes. This infers varying degrees of travel inconvenience potentially experienced by households when seeking healthcare.

Regarding transportation modes, a majority, comprising 83.56%, did not utilize public transport, suggesting a reliance on private means of transportation. Additionally, a significant majority, 94.14%, returned home using the same mode of transport, indicating consistency in transportation choices.

Furthermore, the data reveals insights into social dynamics, with only 12.38% of individuals being accompanied by a companion to the PHC. This indicates that for the majority, healthcare-seeking behaviour occurs independently.

Table 4 offers insights into various time-related aspects concerning individuals’ interactions with PHCs and related responsibilities.

**Table 4.** Factors influencing time management and work obligations during visits to PHCs.

Variables	Categories	Frequency (N = 1502)	Percentage (%)
**Time spent in the PHC (waiting time & consultation time)**	5 and less than 5 minutes	129	8.59
	More than 5 minutes & less than 10 minutes	122	8.12
	More than 10 minutes & less than 20 minutes	254	16.91
	More than 20 minutes & less than 60 minutes	964	64.18
	More than 60 minutes	33	2.20
**Time taken from paid work to come to the PHC (minutes)**	More than 10 mins & less than 30 mins	4	6.35
	More than 30 mins & less than 60 mins	21	33.33
**Number of days in a week, an individual works**	Less than 4 days	4	3.51
	More than 4 days & less than 5 days	39	34.21
	More than 5 days & less than 7 days	71	62.28
**Number of hours in a week, on average, the individual works (hours)**	10 hours and less than 10 hours per week	44	38.60
	More than 10 hours and less than 20 hours per week	6	5.26
	More than 20 hours and less than 40 hours per week	37	32.46
	More than 40 hours and less than 72 hours per week	27	23.68
**Time taken from work to visit the Primary Health Center (minutes)**	10 minutes or less	27	23.68
	More than 10 mins & less than 30 mins	33	28.95
	More than 30 mins & less than 60 mins	38	33.33
	More than 60 mins & less than 120 mins	16	14.04
**Time spent by the companion both in travel time and time spent at the PHC (minutes)**	10 minutes or less	50	26.88
	More than 10 mins & less than 30 mins	41	22.04
	More than 30 mins & less than 60 mins	74	39.78
	More than 60 mins & less than 120 mins	21	11.29
**Amount of time taken off from paid work to accompany the individual to the PHC (minutes)**	10 minutes or less	149	80.11
	More than 10 mins & less than 30 mins	6	3.23
	More than 30 mins & less than 60 mins	13	6.99
	More than 60 mins & less than 120 mins	12	6.45
	More than 120 mins & less than 300 mins	6	3.23
**Time spent looking after the children/dependents by the caregiver when the individual visited the PHC**	15 minutes or less	22	34.92
	More than 15 mins & less than 30 mins	16	25.40
	More than 30 mins & less than 60 mins	21	33.33
	More than 60 mins & less than 120 mins	4	6.35

Firstly, concerning the time spent at the PHC, the majority of individuals, constituting 64.18%, reported durations of more than 20 minutes but less than 60 minutes, emphasizing potentially significant waiting and consultation times. Moreover, 8.12% and 16.91% experienced shorter durations, while a smaller proportion, 2.20%, endured waits exceeding 60 minutes.

In terms of time allocation from paid work to visit the PHC, there’s a distribution across various durations, with 33.33% spending more than 30 minutes but less than 60 minutes, indicating potential disruptions to work schedules for healthcare visits.

Furthermore, data regarding work schedules reveals that the majority, comprising 62.28%, work more than 5 days but less than 7 days per week, underscoring potential challenges in balancing work commitments with healthcare needs.

Regarding time spent by companions, there’s variability, with 39.78% spending more than 30 minutes but less than 60 minutes, possibly reflecting the support provided by companions in accompanying individuals to PHCs.

Moreover, data on time taken off from paid work to accompany individuals to PHCs highlights that a significant majority, at 80.11%, reported durations of 10 minutes or less, indicating minimal disruptions to work for caregiving responsibilities.

Lastly, concerning caregiving responsibilities, caregivers spent varying durations looking after children/dependents during individuals’ visits to PHCs, with 33.33% spending more than 30 minutes but less than 60 minutes, reflecting the impact of healthcare visits on caregiving duties.

Overall, these insights shed light on the time-related challenges and dynamics individuals and their companions face when accessing healthcare services, highlighting areas where interventions or improvements may be necessary to streamline processes and reduce burdens on individuals and their support networks.

Table 5 presents a detailed overview of various time-related factors associated with individuals’ engagements with PHCs and their corresponding duties.

**Table 5.** Time-related challenges that the individuals and their companions encounter when accessing healthcare services.

Variables	Mean	Median	Range	SD
**Time taken to travel from home to the PHC (minutes)**	16.12	15	70	9.84
**Time taken from paid work to come to the PHC (minutes)**	52.52	60	120	45.18
**Time spent by the companion both in travel time and time spent at the PHC (minutes)**	36.18	40	120	27.44
**Amount of time taken off from paid work to accompany the individual to the PHC (minutes)**	62.05	47.50	299	67.13
**Time spent looking after the children/dependents by the caregiver when the individual visited the PHC (minutes)**	30.30	30	120	28.03
**Time taken from work to visit the Primary Health Center (minutes)**	41.71	30	120	35.81

For the duration of travel from home to the PHC, the mean time is 16.12 minutes, with a median of 15 minutes, indicating generally consistent travel times for most individuals. However, there is notable variability, with travel durations ranging from 0 to 70 minutes, and a standard deviation of 9.84 suggests moderate dispersion around the mean.

Regarding the time taken from paid work to reach the PHC, the mean duration is higher at 52.52 minutes, with a median of 60 minutes, reflecting potentially longer commutes for those traveling from their workplaces. The range spans from 0 to 120 minutes, indicating diverse commuting times, with a considerable standard deviation of 45.18.

Companions’ time commitments, including travel and time spent at the PHC, show a mean duration of 36.18 minutes, with a median of 40 minutes, suggesting moderate consistency. However, there is variability, with durations ranging from 0 to 120 minutes and a standard deviation of 27.44.

Individuals taking time off from work to accompany others to the PHC experience a mean duration of 62.05 minutes, with a median of 47.50 minutes, highlighting significant disruptions to work schedules. The range is wide, from 0 to 299 minutes, with a considerable standard deviation of 67.13.

Lastly, caregivers spend an average of 30.30 minutes looking after children/dependents during PHC visits, with a median of 30 minutes, showcasing consistent caregiving responsibilities. Variability exists, with durations ranging from 0 to 120 minutes and a standard deviation of 28.03.

Table 6 presents detailed insights into the various costs and time implications associated with patient travel, companion expenses, childcare, and productivity losses related to visits to the PHC.

**Table 6.** Patient’s comprehensive expenses for rural PHC treatment access.

Variables	Mean	Median	Range	SD
**Patient travel costs**				
Cost of one-way fare if traveled by public transport (INR)	55.34	20	20 - 500	2064.14
Cost of one-way taxi fare (INR)	58.46	50	50 - 198	37.64
Cost of tolls if travel by private car or motorbike (INR)	52.42	50	50 - 199	39.94
**Patient time costs**				
Amount of earnings lost due to time taken off work to go to the PHC (INR)	99.20	50	50 -500	129.35
**Companion costs**				
Cost of one-way fare if the companion traveled by public transport with the patient (INR)	54.60	50	50 -298	56.79
**Childcare and other dependent costs**				
Amount paid to that person to look after children/dependents when the individual visited the PHC (INR)	3.66	0	0 - 20	8.04
**Productivity losses**				
Number of days a week individual works (days)	5.06	6	5 - 6	0.74
Number of hours a week, on average the individual works (hours)	27.23	34	10 - 70	18.50

The average one-way fare for public transport was 55.34 INR (median: 20 INR, range: 20–500 INR), while the cost of a one-way taxi fare averaged 58.46 INR (median: 50 INR). Additional travel costs, such as tolls for private vehicles, had a mean value of 52.42 INR. The earnings lost due to time off work to visit the PHC averaged 99.20 INR, with a median loss of 50 INR. Companion travel costs averaged 54.60 INR, and childcare costs were minimal, with a mean of 3.66 INR. Productivity losses indicate that individuals worked an average of 5.06 days per week (median: 6 days) and 27.23 hours per week (median: 34 hours).

Table 7 presented the multivariable regression analysis to understand the relationship between various socio-economic factors and Total Healthcare Costs (THC) incurred for HPV screening. The model explained 5.9% of the variance (R ^2^ = 0.059, Adjusted R ^2^ = 0.056), indicating that the included predictors had a modest effect on THC. The Durbin-Watson statistic (1.705) suggested no strong autocorrelation in the residuals, and Variance Inflation Factors (VIF < 1.3) confirmed no severe multicollinearity among variables.

**Table 7.** Factors impacting total healthcare costs: Insights from Regression analysis.

Variable	B (Unstandardized Coefficient)	SE (Standard Error)	Beta (Standardized Coefficient)	t	p-value	VIF
** (Constant)**	-250.45	78.92	—	-3.17	0.002**	—
**Employment (1=Employed)**	65.78	16.45	0.098	3.99	0.000**	1.055
**Distance to PHC (km)**	-0.285	0.690	-0.009	-0.41	0.680	1.003
**Education (years)**	108.45	19.87	0.150	5.46	0.000**	1.222
**Income (INR)**	0.000	0.000	0.075	2.89	0.004**	1.113
**Travel Time (minutes)**	5.98	1.24	0.122	4.81	0.000**	1.052
**Companion Accompanied (1=Yes)**	109.87	36.91	0.075	2.98	0.003**	1.023
**Age (years)**	0.94	1.39	0.017	0.68	0.497	1.102

Among the key findings, employed women spent ₹65.78 more on healthcare costs than unemployed women (p < 0.001). Higher education levels were associated to increased costs, with each additional year of education increasing THC by ₹108.45 (p < 0.001). Income also had a small but significant impact, with higher income levels slightly increasing THC (p = 0.004).

Travel time significantly affected costs, with each additional minute increasing THC by ₹5.98 (p < 0.001). Women who traveled with a companion incurred additional ₹109.87 expenses than those who traveled alone (p = 0.003). However, distance to the PHC (₹-0.285, p = 0.680) and age (₹0.94, p = 0.497) were not significant cost factors.

## Discussion

The study emphasizes the significant impact of socioeconomic factors on healthcare accessibility and affordability. The above findings highlight those indirect costs, such as productivity loss and transportation expenses, substantially contribute to the economic burden of HPV screening. Variables like employment status, educational attainment, and income level are pivotal in determining the financial strain associated with accessing HPV screening services. Those with higher socioeconomic status typically face fewer obstacles due to their greater financial means and enhanced access to healthcare facilities. The specific finding that employment status is a significant predictor of the economic hurdles in accessing HPV screening in rural India highlights the intricate interplay between socioeconomic factors and healthcare utilization. This observation is in line with the research conducted by Srivatsa et al., which suggests that women hailing from households with a higher income are significantly more inclined to undergo cervical cancer screening compared to those from lower-income households. ^20^^,^^21^ Additional studies such as Kaneko, 2018, and Keetile et al., 2021 have similarly argued that disadvantaged households are often less informed and thus less likely to prioritize cervical cancer screening. ^22^^,^^23^

A notable finding underlines the influence of travel-related variables on overall expenses. Extended travel duration to the PHC and having a companion during PHC visits are associated with increased total costs. These results are consistent with prior research conducted by Rocque (2019) and Kornelson (2021), ^24^^,^^25^ highlighting the significant contribution of travel-related expenses, to the economic burden experienced by individuals accessing HPV screening in rural areas. This observation is further supported by Wu et al. (2020) and Srinath et al (2023). ^26^^,^^27^ Addressing transportation barriers and providing assistance for travel expenses could prove instrumental in easing the economic burden on vulnerable populations. ^28^^,^^29^

Sriram et al. emphasized the role of healthcare efficiency in reducing patient costs. Longer wait times at PHCs increase total expenses, highlighting the need for streamlined processes and better resource management. Enhancing infrastructure and implementing efficient appointment systems can improve HPV screening accessibility while lowering costs. Their study also found that for-profit hospitals have shorter wait times, attracting wealthier patients. To ensure equitable access, public hospitals must reduce delays. Addressing sociodemographic and community factors can further enhance screening uptake within local healthcare settings. ^30^^–^^32^

Initiatives to enhance access to HPV screening should not only address geographical barriers but also consider the socioeconomic determinants that may deter individuals from seeking preventive care. By addressing these disparities, policymakers and healthcare providers can strive toward ensuring equitable access to vital healthcare services, thereby alleviating the burden of preventable diseases like cervical cancer in rural India and beyond. ^4^^,^^15^^,^^33^

The findings of the study have important policy implications for cervical cancer prevention efforts in rural India. Policy interventions aimed at improving employment opportunities, promoting education, and enhancing transportation infrastructure can help alleviate the socioeconomic barriers to accessing HPV screening services. Additionally, targeted financial assistance programs for low-income individuals and those living in remote areas can help reduce the economic burden associated with seeking healthcare services. ^11^^,^^34^

The study acknowledges several limitations, such as its cross-sectional design and potential confounding factors. Future research could explore longitudinal data to assess the long-term economic impact of accessing HPV screening. Moreover, qualitative studies could provide deeper insights into the lived experiences of individuals accessing HPV screening services and the factors influencing their decision-making processes.

In conclusion, recognizing the key socioeconomic factors and travel-related expenses that impact total healthcare costs, implementing strategies to lower travel costs and alleviate financial barriers especially for lower-income and unemployed women could enhance the accessibility and affordability of screening, thereby improving public health outcomes.

## Ethics approval and consent to participate

Institutional Ethical approval was obtained from the Institutional Review Board of Ohio University on 05.17.2023. IRB project 23-E-101, titled ‘Supply-side and Demand-side Barriers to Access HPV Screening and the Cost-effectiveness analysis of Human Papilloma Virus (HPV) Screening for the Prevention of Cervical Cancer Screening in India. Ohio University’s Institutional Review Board deemed it exempt from review since no interventions were carried out.

Informed written consent was obtained from all study participants before data collection.

## Consent for publication

Not applicable.

## Data Availability

**Harvard Dataverse**: Demand-side Barriers and Economic Burden in Accessing Human Papillomavirus Screening for Cervical Cancer Prevention in Rural India: Evidence from a Cross-sectional Study, https://doi.org/10.7910/DVN/H9DB7B. ^35^ This project contains the following underlying data:
- Microsoft Excel Spreadsheet Data are available under the terms of the Creative Commons Zero “No rights reserved” data waiver (CC0 1.0 Public domain dedication) Harvard Dataverse: Demand-side Barriers and Economic Burden in Accessing Human Papillomavirus Screening for Cervical Cancer Prevention in Rural India: Evidence from a Cross-sectional Study, https://doi.org/10.7910/DVN/H9DB7B. ^35^ This project contains the following extended data:
- Questionnaire Data are available under the terms of the Creative Commons Zero “No rights reserved” data waiver (CC0 1.0 Public domain dedication).
