# Supplementary material for: Demand-side barriers and economic burden in accessing Human Papillomavirus screening for cervical cancer prevention in rural India: Evidence from a cross-sectional study
Source: F1000Res. 2025 Feb 17;13:630. Originally published 2024 Jun 13. [Version 2] doi: 10.12688/f1000research.150361.2 (PMC12134725; doi:10.12688/f1000research.150361.2)
Supplement: Peer review history

# Reviewer report: Reviewer response for version 2

**DOI:** 10.5256/f1000research.177813.r385439

**Published:** 2025-06-03

**Authors:** Inge Dhamanti

**Affiliations:** 1 Universitas Airlangga, Surabaya, Indonesia

The discussion and conclusion are too short; the authors could add some actionable steps or practical recommendations for relevant stakeholders, such as policymakers, healthcare providers, and community organization.  

 The authors need to acknowledge the limitations of the study or methodological constraints in the study and also suggest areas for future research. 

Is the work clearly and accurately presented and does it cite the current literature?

Yes

If applicable, is the statistical analysis and its interpretation appropriate?

Yes

Are all the source data underlying the results available to ensure full reproducibility?

Yes

Is the study design appropriate and is the work technically sound?

Yes

Are the conclusions drawn adequately supported by the results?

Partly

Are sufficient details of methods and analysis provided to allow replication by others?

Yes

Reviewer Expertise:

Public Health, Safety and Quality

I confirm that I have read this submission and believe that I have an appropriate level of expertise to confirm that it is of an acceptable scientific standard, however I have significant reservations, as outlined above.

# Reviewer report: Reviewer response for version 2

**DOI:** 10.5256/f1000research.177813.r379656

**Published:** 2025-05-30

**Authors:** Aurelio Cruz-Valdez

**Affiliations:** 1 Instituto Nacional de Salud Pública, Cuernavaca, Mexico

The topic is important. Social and economic determinants are crucial for cancer mortality. The article addresses an essential aspect of identifying the association with economic determinants in a country rife with social contrasts. However, I believe there are several aspects that need to be clarified:

 In the Introduction section:

 1) It is lengthy and imprecise, and the reader loses the thread between the justification and the objective of the study. This situation is straightforward in the first six sentences of this section. We can consider the Andersen model a guide for this section study’s approach.

 2) The argument for the study of aim is not remains identified.

 Methods Section:

 1) Selection of the study population: We solicit to State the method for calculating the sample size. How did you calculate the variability across these many stages? This allows you to observe the internal and external inferences of the results.

 2) What were the selection and exclusion criteria for the study population?

 3) Variable Selection: The authors note a series of “exposure and outcome” variables, but they not show which variables measure each construct in Andersen’s model. By not mentioning them which concepts each variable measures, which would don’t allow us to understand the creation of the statistical model used in the analysis, and the control of confounding variables.

 4) Regarding the management of covariates and biases, this section is unnecessary. Detailing the data quality and the steps taken to address inconsistencies, particularly in measuring the outcome and economic variables, would be better.

 Why they did not create indices for the different variables measuring different dimensions of the same construct, such as economic variables, time management, or the access. This results in too many tables, which prevents the author from integrating the results. It is striking that the model’s prediction is very low (R2 = 5.9%).

 Discussion Section:

 1) The authors should emphasize the findings comparing to other authors and other contexts. This causes that we don’t identify the importance of results, and this section does not contribute new knowledge. We suggest a more thorough review that considers Andersen’s model, since it is which they used in their theoretical framework from the start.

Is the work clearly and accurately presented and does it cite the current literature?

Yes

If applicable, is the statistical analysis and its interpretation appropriate?

Partly

Are all the source data underlying the results available to ensure full reproducibility?

Partly

Is the study design appropriate and is the work technically sound?

No

Are the conclusions drawn adequately supported by the results?

No

Are sufficient details of methods and analysis provided to allow replication by others?

No

Reviewer Expertise:

Public Health; Cervical cancer epidemiology; HPV infection and cancer

I confirm that I have read this submission and believe that I have an appropriate level of expertise to state that I do not consider it to be of an acceptable scientific standard, for reasons outlined above.

# Reviewer report: Reviewer response for version 1

**DOI:** 10.5256/f1000research.164926.r347336

**Published:** 2025-01-29

**Authors:** Shabana Tharkar

**Affiliations:** 1 King Saud University, Riyadh, Saudi Arabia

This research" Demand-side barriers and economic burden in accessing Human Papillomavirus screening for cervical cancer prevention in rural India: Evidence from a cross-sectional study" addresses an important issue of recognizing barriers to cancer screening in the rural sector of a developing country. However, only economic challenges have been addressed. The paper is well- written and the results are well-presented. 

 Major points

- Introduction and rationale are acceptable but the methods need revision.
- Can revise methods according to STROBE
- Please remove outlier in Table 6, (cost of one way fare) and re -run the stats. 
- The paper can be accepted after revision.

Is the work clearly and accurately presented and does it cite the current literature?

Yes

If applicable, is the statistical analysis and its interpretation appropriate?

Yes

Are all the source data underlying the results available to ensure full reproducibility?

Yes

Is the study design appropriate and is the work technically sound?

Yes

Are the conclusions drawn adequately supported by the results?

Yes

Are sufficient details of methods and analysis provided to allow replication by others?

Yes

Reviewer Expertise:

Epidemiology of non communicable diseases

I confirm that I have read this submission and believe that I have an appropriate level of expertise to confirm that it is of an acceptable scientific standard, however I have significant reservations, as outlined above.

# Response

**Published:** 2025-02-01

**Authors:** Shyamkumar Sriram

**Affiliations:** University of South Carolina, USA

The revisions have been made according to the suggestion by Dr. Shabana Tharkar. Methods have been revised according to STROBE. Outlier has been removed in Table 6 and the statistics have been rerun. The discussion section is also updated. Thanks for the feedback and offering to accept the paper after revisions.

# Reviewer report: Reviewer response for version 1

**DOI:** 10.5256/f1000research.164926.r332791

**Published:** 2024-10-29

**Authors:** Ankit Anand

**Affiliations:** 1 Population Research Centre,, Institute for Social and Economic Change, Karnataka, India

The topic is very interesting and relevant, but the analysis does not add much value to the topic.

 Need to provide details on the estimation of the cost and income levels. 

 Introduction is very generic and does not add why this data and analysis was needed. It states a lot of generic statement and facts which need to be inline why such study is required.

 For example: Author stated “only 1.7% of rural women participated in cervical cancer screening according to data from the National Family Health Survey 5 (NFHS-5).” “Addressing these barriers requires a comprehensive approach encompassing policy reforms, targeted interventions, and community engagement strategies to ensure equitable access to cervical cancer screening services and improve the health outcomes of rural women globally” Study may be needed to explore barriers among female in rural areas. Why Author selected Puducherry which is one of the urban Union Territory of India?

 There seems to a lot of reporting bias and This statement make no sense "Public transport one-way fares exhibit a wide range, spanning from 20 INR to 79,999 INR." 70,000 is a lot of cost for public transport in India. Data need to cleaned (check for outliers and validation before analysing it).

 How was the data quality ensured? It needs to be added.

 The analysis and variables are not well though of. There needs a detail description of how the economic related variables were collected and analysed.

 Most of the variables seem to be correlated in table 7. Distance from facility will be co-related with traveling time and cost of traveling. this is obvious. 

 Having a companion significant increase the cost. this is obvious too, hardly a finding

 Why local ethical board not consulted for the study?

 Why the first author is not from India or Puducherry where the study was performed.

 Conclusion is also generic and does not add any value to the research on HPV screening.

Is the work clearly and accurately presented and does it cite the current literature?

Yes

If applicable, is the statistical analysis and its interpretation appropriate?

No

Are all the source data underlying the results available to ensure full reproducibility?

Partly

Is the study design appropriate and is the work technically sound?

Yes

Are the conclusions drawn adequately supported by the results?

Yes

Are sufficient details of methods and analysis provided to allow replication by others?

Partly

Reviewer Expertise:

Health services research, Women's health, Maternal health, Survey research, Socioeconomic aspects of health

I confirm that I have read this submission and believe that I have an appropriate level of expertise to state that I do not consider it to be of an acceptable scientific standard, for reasons outlined above.

# Reviewer report: Reviewer response for version 1

**DOI:** 10.5256/f1000research.164926.r332790

**Published:** 2024-10-25

**Authors:** Santosh Kumari Duppala

**Affiliations:** 1 Lovely Professional University, Phagwara, Punjab, India

The manuscript effectively outlines the accessibility challenges rural populations face in accessing HPV screening services. The discussion on the economic implications of HPV screening for rural residents is compelling. Thorough Research by the authors have conducted a comprehensive review of the literature, demonstrating a deep understanding of the complexities surrounding HPV screening in rural settings. The manuscript is well-organized and clearly written, making complex information accessible to a broad audience. The authors have done an excellent job of presenting their findings.

 The authors offer innovative and practical solutions to address the economic and travel barriers faced by rural populations, showcasing their forward-thinking approach to public health challenges. Strong Evidence Base where the authors have backed their claims with robust data and evidence, enhancing the credibility of their findings and recommendations. "The statistical analysis of the economic burden related to HPV screening is insightful and provides a nuanced understanding of the challenges rural residents face. The discussion section is particularly engaging, as the authors effectively connect their research to real-world implications, demonstrating the importance of addressing HPV screening in rural communities.

 Recommendations for future manuscript:

1. **Comparative Analysis**: "A comparison of HPV screening rates between urban and rural populations would strengthen it. Highlighting these disparities can underscore the need for targeted interventions."
2. **Policy Recommendations**: "It would be beneficial give recommendations on policy changes that could alleviate the economic and travel burdens associated with HPV screening in rural areas in future manuscript.
3. **Telehealth Solutions**: "A small suggestion of tele-health as a potential solution for HPV screening in rural communities could be added in future perspectives and a practical approach to overcome travel and cost barriers."

Is the work clearly and accurately presented and does it cite the current literature?

Yes

If applicable, is the statistical analysis and its interpretation appropriate?

Yes

Are all the source data underlying the results available to ensure full reproducibility?

Yes

Is the study design appropriate and is the work technically sound?

Yes

Are the conclusions drawn adequately supported by the results?

Yes

Are sufficient details of methods and analysis provided to allow replication by others?

Yes

Reviewer Expertise:

Cancer biology, Next generation sequencing technologies,  Bioinformatics, Cervical cancer and HPV, Molecular biology, Microbiology.

I confirm that I have read this submission and believe that I have an appropriate level of expertise to confirm that it is of an acceptable scientific standard.
